# Non-genitourinary *Ureaplasma urealyticum* infections in solid organ transplant recipients: a case report and literature review

**DOI:** 10.1186/s12879-025-12223-4

**Published:** 2025-11-27

**Authors:** Fang Wang, Qing Zhan, Hong Zhu, Lei Guo, Lingbin Shu, Tingting Qu

**Affiliations:** 1 Infection Control Department, The First Affiliated Hospital, Zhejiang University School of Medicine, Hangzhou, 310003 China; 2 Department of Cardiovascular Surgery, The First Affiliated Hospital, Zhejiang University School of Medicine, Hangzhou, 310003 China; 3 Department of Laboratory Medicine, The First Affiliated Hospital, Zhejiang University School of Medicine, Hangzhou, 310003 China; 4 State Key Laboratory for Diagnosis and Treatment of Infectious Diseases, National Clinical Research Center for Infectious Diseases, Collaborative Innovation Center for Diagnosis and Treatment of Infectious Diseases, The First Affiliated Hospital, Zhejiang University School of Medicine, Hangzhou, 310003 China

**Keywords:** *Ureaplasma urealyticum*, Solid organ transplantation, Infection, Omadacycline

## Abstract

**Background:**

*Ureaplasma urealyticum*, a commensal organism, is potentially pathogenic. In solid organ transplant recipients, non-genitourinary *U. urealyticum* infection is associated with an increased risk of graft failure or death.

**Results:**

We reported a case of mediastinitis caused by *U. urealyticum* in a heart transplant recipient and reviewed 13 other cases previously described. Among the 14 patients, 3 were female and 11 were male, with a median age of 61 years. The median time to symptom onset was 9 days after surgery. Among the 10 documented cases with reported symptoms, altered mental status and hyperammonemia syndrome occurred in 9 cases, whereas our patient manifested persistent fever. Both culture and molecular diagnostics were employed in the reviewed cases, with molecular methods predominating. *U. urealyticum* was difficult to cover with initial empirical antibiotic therapy; the patient in this study improved after omadacycline antimicrobial therapy and was successfully discharged following subsequent management. Regarding clinical outcomes, four patients died or withdrew from treatment, while targeted therapy duration among surviving patients ranged from 9 days to 4 weeks.

**Conclusions:**

There is a possibility of severe *U. urealyticum* infection in patients with immunodeficiency after organ transplantation. Monitoring ammonia levels, utilizing rapid diagnostics, and initiating prompt treatment are all crucial to improving prognosis and reducing severe nerve damage, organ dysfunction, and mortality.

**Clinical trial number:**

Not applicable.

**Supplementary Information:**

The online version contains supplementary material available at 10.1186/s12879-025-12223-4.

## Introduction

*Ureaplasma urealyticum* (*U. urealyticum*), a member of the *Mycoplasma* family, is among the smallest free-capable self-replicating living-forms [1]. It frequently colonizes the human urogenital and respiratory tracts; asymptomatic urogenital colonization occurs in about 10%-40% of men and 20%-80% of women [2, 3]. Besides genital tract infections, *Ureaplasma* is also associated with infertility, adverse pregnancy outcomes, and diseases in the newborn [1, 4]. Although relatively uncommon, *U. urealyticum* can cause non-genitourinary tract infections (e.g., of the respiratory tract, joints, central nervous system, and bloodstream), particularly in high-risk populations like those with cardiopulmonary compromise or immunosuppression [5, 6]. A key mechanism of its pathogenicity is urease production, which converts urea to ammonia. In systemic infection, this can lead to a critical disruption of ammonia homeostasis, resulting in hyperammonemia syndrome (HS) [7]. This condition, marked by elevated serum ammonia and neurological manifestations like seizures, can cause irreversible brain damage and is reported in 0.5–4.1% of lung transplant recipients [8].

Previously, *U. urealyticum* was often overlooked due to diagnostic limitations. Improved molecular and culture methods have now revealed a higher than previously appreciated prevalence of its non-genitourinary infections [2]. Solid organ transplantation (SOT) is the optimal treatment for end-stage organ failure, yet postoperative infection control critically influences transplant success and patient survival [9, 10]. *U. urealyticum*’s intrinsic resistance to β-lactams and synergy with immunosuppressants enable rare surgical-site invasive infections (e.g., bacteremia, meningitis) [11, 12]. Its role in post-transplant infections is increasingly recognized, as reflected by numerous recent reports [6, 13]. Due to diagnostic challenges and rising antimicrobial resistance, *U. urealyticum* infection is often identified late, leading to treatment failure and potentially graft loss [14, 15]. Thus, early detection and precise therapy are crucial.

Here, we report a case of mediastinitis caused by *U. urealyticum* in a heart transplant recipient, which was diagnosed successfully and treated at an early stage. Additionally, we present a literature review of non-genitourinary tract *U. urealyticum* infections occurring within the first year after solid organ transplantation.

## Case report

A 60-year-old woman with a history of dilated cardiomyopathy and a permanent atrial-ventricular sequential pacemaker implantation was admitted via the emergency department due to refractory heart failure and cardiogenic shock. Her main clinical manifestations included chest tightness, shortness of breath, and bilateral lower limb edema. The patient suddenly lost consciousness and suffered cardiac arrest in the early hours of the following day. After cardiopulmonary resuscitation, she was transferred to the intensive care unit (ICU). During her stay in the ICU, the patient experienced another episode of ventricular tachycardia. Following electrical cardioversion, vasoactive medications were administered to maintain her blood pressure. However, an echocardiogram revealed partial myocardial fibrosis and impaired systolic function in both ventricles, with a left ventricular ejection fraction (LVEF) of less than 20%. Consequently, veno-arterial extracorporeal membrane oxygenation (VA-ECMO) was employed for circulatory support. Computed tomography (CT) scan showed bilateral pulmonary exudates and multifocal inflammation. The relevant blood test showed high inflammatory parameters (C-reactive protein (CRP) 141.89 mg/L, procalcitonin (PCT) 0.65 ng/mL) and serum creatinine 137 µmol/L. The routine urine test showed positive occult blood (+++), red blood cells 2037.40/µL, and a few bacteria, but the leukocyte esterase was negative. The urine culture detected a small quantity of *Enterococcus faecium*, and antimicrobial therapy has not been administered. The results of blood and sputum cultures and the screening of carbapenem-resistant *Enterobacteriaceae* (CRE) bacteria in feces were all negative. Piperacillin-tazobactam (4.5 g q8h) was empirically initiated to target the pneumonia. The patient’s condition stabilized by the third day post-resuscitation. However, due to persistently poor cardiac pump function, the decision was made to proceed with cardiac transplantation. The donor was a 46-year-old male who was declared brain dead following a cerebrovascular accident and for whom consent for organ donation was obtained from his family. Routine cultures from the donor blood, cardiac preservation solution, and tissue all yielded negative results. Preoperatively, immunosuppressive induction was initiated with intravenous basiliximab (20 mg), and baseline immunosuppression was established with oral mycophenolate sodium enteric-coated tablets (720 mg). Additionally, piperacillin-tazobactam (4.5 g) was administered as standard empirical prophylaxis. The subsequent operation, which lasted 5 h and 38 min, was successful. Intraoperatively, the patient received an additional dose of piperacillin-tazobactam (4.5 g) and an intravenous injection of methylprednisolone (1000 mg). Upon completion, drainage tubes were placed in the pericardium, mediastinum, and right thorax. The postoperative immunosuppressive regimen consisted of tacrolimus (target level: 10–12 ng/mL tapered to 5–8 ng/mL), mycophenolate sodium (initially 720 mg twice daily and tapered), and intravenous methylprednisolone with rapid tapering from a starting dose of 80 mg every 8 h. Due to elevated postoperative CRP (89.11 mg/L) and PCT (3.72 ng/mL), the antibiotic regimen was adjusted to meropenem (1 g q8h) and daptomycin (0.5 g daily) for anti-infective treatment.

On postoperative day (POD) 5, the patient developed fever (maximum 39.4 °C), and CRP was 25.61 mg/L after ECMO discontinuation. On POD 7, the relevant blood test revealed an elevated white blood cell count with CRP at 44.66 mg/L but normal PCT levels. Chest CT revealed bilateral pulmonary exudative lesions with focal consolidation, left pleural effusion complicated by left lower lobe atelectasis, accompanied by minimal anterior mediastinal exudation and pericardial effusion. The antimicrobial was changed to colistin (0.15 g q12h), ceftazidime-avibactam (0.25 g q8h) and caspofungin (50 mg daily), but the patient remained febrile. However, the culture results for blood and urine were all negative. On POD 9, all the drainage tubes were removed with concurrent collection of mediastinal drainage fluid for routine bacterial and *Mycoplasma* cultures (*Mycoplasma* IST-II, bioMérieux, France). The *Mycoplasma* culture results showed heavy growth of *U. urealyticum*, while the routine bacterial culture was negative. On POD 11, mediastinitis was suspected to be caused by *U. urealyticum*, despite normal blood ammonia (20.8 µmol/L). The treatment was initiated with omadacycline (100 mg daily, doubling the initial dose) and azithromycin (0.5 g daily). On POD 13, the fever quickly subsided and the levels of inflammatory indicators decreased (CRP 18.23 mg/L). Moreover, the patient’s sputum volume decreased, and CT showed that the exudation in both lungs was essentially improved. In subsequent days, secretions specimens obtained from drainage tube exit sites returned negative for *Mycoplasma* culture, and then the use of azithromycin was discontinued. Upon attainment of clinical stability, omadacycline therapy was terminated with subsequent transfer to a general ward. The total duration of antibiotic therapy was 4 days with azithromycin, followed by 9 days with omadacycline. Table 1 shows the results of relevant blood tests during the hospital stay. After subsequent treatment, the patient recovered completely and was discharged from the hospital, and maintained a favorable clinical status throughout the 18-month outpatient follow-up period.

**Table 1:** Results of relevant blood investigations at infection onset, after 9 days of antibiotic treatment, and the day before transfer to a general ward

Variable	Reference range in our hospital	The point of infection onset (POD 9)	Nine days after targeted treatment (POD 20)	The day before transfer to a general ward (POD 25)
Body temperature (℃)	36.3–37.2	38.8 ± 2	36.5	36.8
Leukocyte count (10^9^/L)	4.0–10.0	30.7	16.01	10.78
Absolute neutrophil count (10^9^/L)	2.0–7.0	28.95	15.26	9.79
C-reactive protein (mg/L)	0.00–8.00	35.24	11.3	3.87
Procalcitonin (ng/mL)	0.00-0.50	0.4	0.78	0.26
Creatinine (µmol/L)	41–81	199	78	60
Urea (µmol/L)	3.10–8.80	29.29	5.79	4.64
blood ammonia (µmol/L)	9.0–30	20.8(POD12)	/	/

### Literature review

A search of the PubMed and MEDLINE databases was performed until 25 April 2025, including studies written in English. The search employed the following target terms: “transplant”, “*Ureaplasma*”, “*Ureaplasma Urealyticum*”. This search yielded 117 English articles. Inclusion criteria for the literature were: case reports or case series providing detailed clinical information and extractable complete case data. The exclusion criteria included: duplicate reports, animal experiments, clinical trials, secondary analyses of literature, and literature where the original text or full text could not be located. Cases within the eligible literature were screened based on the following criteria: (1) Only cases of *U. urealyticum* infection occurring after solid organ transplantation were included. (2) Infection diagnosis had to be clearly defined, supported by confirmatory culture results or molecular test results. (3) Cases with mixed infections predominantly involving *M. hominis* were excluded. (4) Infections localized to the genitourinary system were excluded. (5) Cases where infection occurred more than one year post-transplantation were excluded. The final review included ten eligible studies that described 13 cases. The case reported here has also been included in the literature analysis and review [16–25]. The general characteristics and outcomes of all included cases are summarized in Table 2.

**Table 2:** Summary of clinical data of recipients with *Ureaplasma urealyticum* infection within one year post-solid organ transplantation

Cases	Pub. Year	Age (years), Sex	Country	Previous Disease(s)	Transplanted Organs	Main Symptom(s)	Presence of HS	Infection Time	Positive Sample	Species Isolated (Method)	Empirical Treatment	Targeted Treatment (Duration)	Outcome
1 [13]	2023	NM, Male	Australia	COPD	Lung	NM	NM	POD 14	BALF	UU (culture)	NM	DOX and MXF (2 weeks)	Continue the treatment
2 [13]	2023	NM, Male	Australia	COPD	Lung	NM	NM	POD 14	BALF	UU (culture)	NM	DOX (2 weeks)	Recovery
3 [13]	2023	NM, Male	Australia	ILD	Lung	NM	NM	POD 5	BALF	UU (culture)	NM	DOX and CIP (2 weeks)	Death
4 [13]	2023	NM, Male	Australia	ILD	Lung	NM	NM	POD 5	BALF	UU (culture)	NM	DOX (4 weeks)	Recovery
5 [14]	2022	64, Male	USA	IPF	Lung	Confused and disoriented	YES	POD 3	BALF	UU (PCR)	LEV	DOX and LEV (2 weeks)	Improvement
6 [15]	2020	51, Female	Germay	RA	Lung	Agitation and disoriented	YES	POD 14	BALF and MLN	UU (PCR)	NM	DOX (NM)	NM
7 [16]	2023	67, Female	China	PIF	Lung	Intractable epileptic seizures	YES	POD 16	BALF	UU and UP (mNGS)	CSL, VRC	AZM (NM)	Withdrawing treatment
8 [17]	2018	62, Male	USA	COPD	Lung	Lethargic and comatose	YES	POD 28	Pleural fluid and BALF	*Ureaplasma* (16 S rRNA PCR, sequence and culture)	PTZ, VA, CRO and VRC	DOX (NM)	Improvement
9 [18]	2022	67, Male	Canada	IPF	Lung	Agitation and decreased level of consciousness	YES	POD 5	BALF	UU (PCR)	PTZ	DOX (2 weeks)	Improvement
10 [19]	2015	44, Male	USA	PS	Lung	Mental status changes	YES	POD 7	Blood, tissue	UU (PCR)	SMZ-TMP and VRC	AZM (5 days)	Death
11 [20]	2017	44, Male	USA	d-TGA	Heart	Nausea and obtunded	YES	POD 3	Serum	UU (PCR)	AZM and DOX	DOX and LEV (NM)	Improvement
12 [21]	2024	65, Male	China	FSGS	Kidney	Pain, vomitus, apathy and dullness	YES	POD 77	Blood	UU (mNGS)	LZD	LEV and MIN (NM)	Death
13 [22]	2008	38, Male	Germay	IN	Kidney	Cephalgias and episode seizure	YES	NM	CSF	UU (16 S rRNA PCR, sequence and culture)	MEM and TOB	DOX, MXF and CM (NM)	Recovery
14	PR	60, Female	China	DCM	Heart	Persistent fever	NO	POD 9	MDF	UU (culture)	CAZ-AVI, CST and CAS	AZM (4 days) and omadacycline (9 days)	Improvement

Abbreviations: Pub. Year Publication, Year PR present report, NM not mentioned, COPD chronic obstructive pulmonary disease, ILD interstitial lung disease, IPF idiopathic pulmonary fibrosis, PIF pulmonary interstitial fbrosis, RA rheumatoid arthritis, PS pulmonary sarcoidosis, d-TGA dextro-transposition of the great arteries, FSGS focal segmental glomerulosclerosis, IN interstitial nephritis, DCM dilated cardiomyopathy, HS Hyperammonemia syndrome, POD postoperative day, BALF cerebrospinal fluid, MLN mediastinal lymph nodes, CSF cerebrospinal fluid, MDF mediastinal drainage fluid, PCR polymerase chain reaction, mNGS metagenomic next-generation sequencing, UU *Ureaplasma urealyticum*, UP *Ureaplasma parvum*, DOX doxycycline, MXF moxifloxacine, CIP ciprofloxacin, LEV levofloxacin, CSL cefoperazone-sulbactam, VRC voriconazole, AZM azithromycin, PTZ piperacillin-tazobactam, VA vancomycin, CRO ceftriaxone, SMZ-TMP sulfamethoxazole/trimethoprim, LZD linezolid, MIN minocycline, MEM meropenem, TOB tobramycin, CM chloramphenicol, CAZ-AVI ceftazidime avibactam, CST colistin, CAS caspofungin

The data summarized in Table 2 indicate that among solid organ transplant recipients with confirmed *U. urealyticum*–positive results included in this analysis, 10 cases were lung transplant recipients (10/14, 71.43%), 2 were heart transplant recipients, and 2 were kidney transplant recipients. Of these, 3 were female and 11 were male (11/14, 78.57%). The median age was 61 years (range: 38 to 67 years), with 4 patients aged ≤ 51 years and the remainder aged ≥ 60 years. Among the 10 cases that reported clinical symptoms, only the heart transplant recipient described in our case presented with persistent fever following *U. urealyticum* infection; the remaining cases primarily exhibited central nervous system manifestations and developed hyperammonemia syndrome (9/10, 90.00%). The median time to symptom onset post-transplantation was 9 days (range: 3 to 77 days). Both culture and molecular diagnostics played critical roles in *U. urealyticum* diagnosis. Specifically, five cases (5/14, 35.71%) were diagnosed using culture-based methods, 7 cases (7/14, 50.00%) were confirmed through molecular diagnostics, and 2 cases (2/14, 14.29%) utilized both detection methods. The primary sources of positive samples included bronchoalveolar lavage fluid (9/14, 64.29%), blood or serum (3/14, 21.43%). Other sources included tissue, mediastinal lymph nodes, cerebrospinal fluid, Pleural fluid and mediastinal drainage fluid. Among the 9 patients for whom empirical antibiotic therapy was documented, the majority received regimens that did not cover *U. urealyticum* (7/9, 77.78%). In contrast, targeted therapies primarily consisted of tetracyclines (doxycycline, minocycline), macrolides (azithromycin), fluoroquinolones (ciprofloxacin, levofloxacin, moxifloxacin), and novel tetracyclines (omadacycline). Regarding clinical outcomes, 4 patients died or discontinued treatment, 5 showed clinical improvement, 3 achieved complete recovery, and 1 was reported as continuing treatment without a clearly defined outcome. The duration of targeted therapy among surviving patients ranged from 9 days to 4 weeks.

### Statistical analysis

All data were analysed using the SPSS statistical software package (version 19; IBM SPSS Statistics). Data of normal distribution were expressed as the mean ± standard deviation (x ± s), and data of abnormal distribution by median and range. The collected count data were evaluated using descriptive statistics, including numbers and percentages.

## Discussion

The pathogenicity of *U. urealyticum*, a widespread genitourinary colonizer, is contingent upon host immune status, with symptomatic infection typically manifesting only in immunodeficient hosts [26, 27]. In patients who undergo organ transplants, graft loss and death may result. A systematic review indicates that this infection significantly increases the risk of hyperinflammation syndrome (HS) and confers a markedly higher mortality (27.27% for *Ureaplasma*-associated HS vs. 5.24% for other causes) [15]. A narrative review further supports the high mortality, reporting a rate of 45.45% in lung transplant recipients [24]. In our study, 30.77% of cases resulted in death or treatment withdrawal, 75% of which involved lung transplants.

In this study, we report a case of mediastinitis caused by *U. urealyticum* in a heart transplant recipient and review the literature on non-genitourinary *U. urealyticum* infections during the initial year following solid organ transplantation. Given the differences in pathogenic mechanisms and antimicrobial susceptibility between *M. hominis* and *U. urealyticum*, cases of co-infection predominantly involving *M. hominis* were excluded from this study. Additionally, considering the cumulative effects of immunosuppression and the high-risk period for opportunistic infections post-transplantation, cases of infection occurring beyond the first year after solid organ transplantation were excluded [28]. Among the 14 cases included in the analysis, the majority were lung transplant recipients (71.43%), with a notably higher incidence of infection compared to kidney and heart transplant recipients. The median age of infected individuals was 61 years. In terms of sex distribution, males predominated, comprising 78.57% of cases. This pattern is likely attributable to the higher prevalence of underlying diseases such as idiopathic pulmonary fibrosis (IPF) and chronic obstructive pulmonary disease (COPD) among male heart and lung transplant recipients. These conditions are more common in males and are often accompanied by baseline risk factors such as a history of smoking, which may contribute to increased susceptibility to postoperative infections [29, 30].

The precise origin of *Ureaplasma* infections remains uncertain, with evidence supporting both donor-derived and endogenous sources. Some reports attribute infections to endogenous reactivation in immunosuppressed recipients, possibly triggered by procedures like urinary catheterization [22, 31]. Immunosuppressive therapy, essential for patients undergoing organ transplantation, renders them susceptible to infections and may foster the dissemination of *U. urealyticum* from the genitourinary tract [6, 27]. However, donor-derived transmission of *Ureaplasma* species has been reported. Data from some studies suggest that transmission of this organism from the donor is a possible mechanism, because donor BAL before the lung procurement was positive for *Ureaplasma* species [22]. Furthermore, a retrospective study further suggests that donor bronchus positivity for *M. hominis* and *U. urealyticum*/*parvum* is a stronger predictor of Mollicute infection than candidate urine testing [17]. A critical limitation across these studies is the lack of molecular typing to confirm clonality and definitively establish the transmission link. Future research is needed to elucidate the specific mechanisms of transmission and the sources of contamination in these situations. In our case, routine donor screening was negative, but this does not rule out a donor source, as standard culture methods frequently fail to detect these fastidious organisms.

Current research and clinical practice have shown that standard microbiological detection methods may not be sufficient for detecting *U. urealyticum* due to the organism’s lack of cell walls. This characteristic prevents visualization under light microscopy with Gram stain and inhibits growth on conventional media [1]. Culture remains the laboratory gold standard for diagnosis, but *U. urealyticum* is not reported in routine microbiological sample testing, as it can only be isolated with a special medium containing urea. This represents a major impediment to its identification using standard culture media and explains the delay in correct diagnosis and treatment in some cases. As shown in Table 1, Cases 1, 2, 3, 4, 8, 13 and 14 utilized culture methods. In recent years, commercially available diagnostic assays with similar methods of identification, antimicrobial susceptibility testing, such as Mycoplasma IST 2 (BioMérieux), have been widely used in clinical practice due to their rapid turnaround (24–48 h) and ease of use. Despite improvements in these kits, traditional culture remains popular in clinical settings due to its low cost and ease of use. However, traditional culture has several limitations, including longer turnaround times than commercial kits, lower sensitivity, and risk of sample contamination. Therefore, molecular methods are increasingly used as complementary or alternative methods for rapid diagnosis. Apart from culture and traditional PCR techniques, new methods with great specificity and sensitivity have appeared, and these include multiplex real-time PCR, loop-mediated isothermal amplification and gold nanoparticle colorimetric detection methods [1]. Cases 5, 6, 8, 9, 10, 11, and 13 employed polymerase chain reaction (PCR), which offers the advantages of speed, high sensitivity, and strong specificity [32]. However, PCR relies on pre-designed primers and can only detect specific targets. In addition, cases 7 and 12 were diagnosed using metagenomic next-generation sequencing (mNGS). Due to their high sensitivity, high specificity and high accuracy, mNGS technologies have emerged as powerful tools for molecular biology research and clinical diagnostic applications [33, 34]. Nonetheless, the implementation of mNGS as a routine clinical diagnostic method remains challenging due to its lack of targeting specificity and high cost. Leveraging the strengths of multiple diagnostic approaches is crucial for the early detection, precise diagnosis and timely targeted treatment of *U. urealyticum* infections.

Empiric antibiotic therapy is directed toward common gram-positive pathogens rather than *Ureaplasma*. Transplant recipients commonly receive perioperative prophylaxis with β-lactams or vancomycin. However, these antibiotics are ineffective against *Ureaplasma* due to its lack of a bacterial cell wall, which is the target of these antibiotics [35, 36]. Furthermore, other commonly considered agents, such as gentamicin and folate antagonists (e.g., trimethoprim/sulfamethoxazole), are also ineffective. In this study, only 22.22% recipients (2/9) had received agents active against *U. urealyticum* before pathogen identification. Effective treatment options may include tetracycline, macrolide and fluoroquinolone antibiotics, although emerging resistance to all of these classes is becoming increasingly concerning [36, 37]. Antibiotic resistance rates of *U. urealyticum* exhibit significant geographic variability, with resistance rates in Asia reported to be higher than those in Western countries [1, 14, 37]. In this case report, the isolate was resistant to fluoroquinolones, tetracycline, and other antibiotics that were tested by the *Mycoplasma* IST 2 kit (S1). Although the IST 2 kit might overstate fluoroquinolone resistance, omadacycline was ultimately selected due to the patient’s critical condition complicated by renal impairment. The patient responded rapidly to the therapy. Omadacycline (9-neopentylaminomethylminocycline) is a novel and promising aminomethylcycline available in both intravenous and oral formulations [38]. Omadacycline had the lowest MIC_90_ among all drugs tested against Mollicutes and its activity was not affected by macrolide, tetracycline, or fluoroquinolone resistance [39]. In addition, patients with hepatic and renal insufficiency, including those with end-stage renal disease undergoing hemodialysis, do not require omadacycline dose adjustments [40].

Targeted antimicrobial therapy is often adjusted based on diagnostic results and the timing of clinical symptom onset. The median onset of clinical symptoms was 9 days post-transplant (from 3 to 77 days), and the isolation or detection of *U. urealyticum* typically occurred after the onset of symptoms. Among the severe infection cases with documented clinical symptoms, recipients in Cases 5–13 developed HS and serious central nervous system symptoms, including agitation, seizures, confusion, and even coma, accounting for 90% of the cases. *U. urealyticum* can induce hyperammonemia syndrome (HS) through the hydrolysis of urea into ammonia, catalyzed by cytoplasmic urease [41]. Patients with HS caused by non-genitourinary infections mainly present nervous system symptoms, often accompanied by brain edema as a typical imaging change. In contrast, the heart transplant recipient reported in the present study exhibited a different clinical presentation, characterized by persistent high-grade fever without neurological abnormalities. Although the patient had preexisting renal impairment due to cardiac arrest prior to transplantation, HS was not observed. This may be attributed to timely targeted treatment. Under immunosuppressive conditions, early manifestations of *U. urealyticum* infection may be obscured by other postoperative symptoms. In heart and lung transplant recipients, this challenge is further compounded by the use of extracorporeal membrane oxygenation (ECMO), which can blunt febrile responses. Moreover, the duration of targeted treatment among surviving patients ranged from 9 days to 4 weeks, and 30.77% (4/13) were reported to have died or withdrawn from treatment, with lung transplant recipients accounting for 75% of these cases. Therefore, the ideal duration of antibiotic treatment for non-genitourinary tract *U. urealyticum* infections following solid organ transplantation varies depending on the site of infection, the recipient’s clinical condition, the pathogen’s resistance profile, and the timing of intervention.

There are several limitations to this study. First, this was a single-center case report with a narrative review of only 14 cases from the literature, therefore, our observations may not be generalizable to all SOT populations. Second, the management, diagnostic platforms, and reporting of ammonia levels were heterogeneous across reports, which limited our ability to perform pooled analysis of risk factors, treatment duration, or outcome predictors. Third, potential donor-derived infections could not be completely ruled out because detailed donor screening was not consistently available in the literature. Finally, long-term relapse data were rarely reported, so the optimal duration of targeted therapy remains uncertain.

In summary, severe *U. urealyticum* infection represents a potential threat to immunocompromised organ transplant recipients. Given the severity of this infection, prompt consultation with clinical bacteriology laboratories and infectious disease specialists is warranted upon suspicion. Based on the present case and a review of the literature, we propose the following pragmatic suggestion for centers performing SOT, especially heart or lung transplants: (i) Pretransplant screening of recipients for *Ureaplasma* should be considered when risk factors for infection are present, such as abnormal urinalysis, urogenital tract infection, or renal dysfunction. (ii) Closely monitor for neurological symptoms with consideration of serum ammonia testing in the early postoperative period; and (iii) in critically ill SOT recipients with early postoperative fever or neurological symptoms, it may be necessary to empirically add one or two *Ureaplasma*-active agents (e.g., doxycycline, azithromycin) or a novel antimicrobial agent (e.g., such as omadacycline) while awaiting specific test results. Currently, there are no guidelines for the management of non-genitourinary *U. urealyticum* infections in SOT recipients. Thus, reporting new cases is useful for sharing successful treatment strategies.

## Supplementary Information

Below is the link to the electronic supplementary material.

Supplementary Material 1

## Data Availability

The original contributions of this study are included in the article/supplementary material section. Further enquiries can be directed to the corresponding authors.

## Abbreviations

AZM: Azithromycin
BALF: Cerebrospinal fluid
BPH: Benign prostatic hyperplasia
CAZ-AVI: Ceftazidime avibactam
CAS: Caspofungin
CIP: Ciprofloxacin
CM: Chloramphenicol
COPD: Chronic obstructive pulmonary disease
CRE: Carbapenem-resistant Enterobacteriaceae
CRO: Ceftriaxone
CRP: C-reactive protein
CSF: Cerebrospinal fluid
CSL: Cefoperazone-sulbactam
CST: Colistin
CT: Computed tomography
DCM: Dilated cardiomyopathy
DOX: Doxycycline
d-TGA: dextro-transposition of the great arteries
FSGS: Focal segmental glomerulosclerosis
HS: Hyperammonemia syndrome
ICU: Intensive care unit
ILD: Interstitial lung disease
IN: Interstitial nephritis
IPF: Idiopathic pulmonary fibrosis
LEV: Levofloxacin
LZD: Linezolid
MDF: Mediastinal drainage fluid
MEM: Meropenem
MIN: Minocycline
MLN: Mediastinal lymph nodes
mNGS: metagenomic next-generation sequencing
MXF: Moxifloxacine
NM: Not mentioned
PCR: Polymerase chain reaction
PCT: Procalcitonin
PIF: Pulmonary interstitial fbrosis
POD: Postoperative day
PR: Present report
PS: Pulmonary sarcoidosis
PTZ: Piperacillin-tazobactam
RA: Rheumatoid arthritis
SMZ-TMP: Sulfamethoxazole/trimethoprim
SOT: Solid organ transplantation
TOB: Tobramycin
UP: Ureaplasma parvum
UU: Ureaplasma urealyticum
VA: Vancomycin
VA-ECMO: Veno-arterial extracorporeal membrane oxygenation
VRC: Voriconazole
