# Supplementary material for: Non-genitourinary Ureaplasma urealyticum infections in solid organ transplant recipients: a case report and literature review
Source: BMC Infect Dis. 2025 Nov 27;25:1827. doi: 10.1186/s12879-025-12223-4 (PMC12752152; doi:10.1186/s12879-025-12223-4)
Supplement: Supplementary file 1 — Supplementary Material 1 [file 12879_2025_12223_MOESM1_ESM.docx]

**The standard empiric antibiotic regimen used at our center post-transplant.**

At our center, the standard empiric postoperative prophylaxis for heart transplantation includes a broad-spectrum β-lactams (typically piperacillin-tazobactam or meropenem), optionally supplemented with an anti-Gram-positive agent (such as daptomycin or vancomycin), guided by local epidemiology and donor/recipient colonization screening. This regimen is subsequently adjusted based on pathogen detection results and inflammatory response indicators

**Table 1. MIC of antibiotics for the *U. urealyticum* isolates by the Mycoplasma IST 2 kit.**

| Antibiotics | Results | MIC (μg/mL) | |
| --- | --- | --- | --- |
| Erythromycin | R | | ≥8 |
| Tetracycline | R | | ≥16 |
| Pristinamycin | R | | ≥4 |
| Doxycycline | R | | ≥8 |
| Clarithromycin | R | | ≥8 |
| Ofloxacin | R | | ≥8 |
| Josamycin | R | | ≥16 |
| Ciprofloxacin | R | | ≥4 |
| Azithromycin | R | | ≥8 |

MIC minimum inhibitory concentration, R resistance, S sensitive, I intermediate
